# Utilization of Alloplastic Prostheses in the Extended Reconstruction of the Temporomandibular Joint: A Review of the Literature

**DOI:** 10.3390/jcm13226748

**Published:** 2024-11-09

**Authors:** Yasser S. Alali, Khaled Al Habeeb, Khaled Al Malhook, Wajdi A. Mohammed (Bin), Sami Alshehri, Bader Fatani

**Affiliations:** 1 Department of Maxillofacial Surgery, College of Dentistry, King Saud University, Riyadh 11545, Saudi Arabia; yalali@ksu.edu.sa (Y.S.A.); kmh-100@hotmail.com (K.A.H.); khalidmhq7@gmail.com (K.A.M.); 2 Department of Oral Medicine and Diagnostic Sciences, College of Dentistry, King Saud University, Riyadh 11545, Saudi Arabia; wbinmohammed@ksu.edu.sa; 3 Department of Biomedical Dental Sciences, College of Dentistry, Imam Abdulrahman Bin Faisal University, Dammam 31441, Saudi Arabia; smalshehri@iau.edu.sa; 4 College of Dentistry, King Saud University, Riyadh 11545, Saudi Arabia

**Keywords:** extended alloplastic temporomandibular joint reconstruction, alloplastic temporomandibular joint replacement, total joint replacement, total joint reconstruction, virtual surgical planning

## Abstract

Extended temporomandibular joint reconstruction (eTMJR) can be described as a refined approach to conventional temporomandibular joint reconstruction (TMJR) designed to address both the articulating components of the TMJ and associated mandibular segmental defects or defects in the skull base. Extended temporomandibular joint reconstruction (eTMJR) combined with the advancement in VSP not only offers improved functional and esthetic outcomes but also signifies a significant leap forward in the realm of TMJR interventions. In comparison to autogenous grafts, alloplastic pro$stheses exhibit superior outcomes concerning MIO, pain management, and dietary functionality, underscoring their potential as the preferred treatment modality. This review article showcases an in-depth exploration of eTMJR, covering its classifications, indications, contraindications, advantages, disadvantages, complications, virtual surgical planning (VSP), criteria for successful alloplastic devices, and the surgical approach.

## 1. Introduction

Extended temporomandibular joint reconstruction (eTMJR) can be described as a modification of conventional TMJR. It involves both articulating components of the TMJ (fossa-condyle complex) and associated mandibular segmental defects and/or defects in the skull base [1]. TMJR treatments can be either autogenous or alloplastic. In autogenous approaches, the most generally used surgical technique is a costochondral graft, particularly preferred for children with growth potential. However, problems such as the morbidity of the donor site, ankylosis, and unpredictable growth behavior of the graft may be encountered. To address these problems, alloplastic materials have been innovated [2].

The largest suppliers of alloplastic TMJ prostheses are Stryker-TMJ Concepts (Ventura, CA, USA) and Zimmer BioMet (Biomet Microfixation, Jacksonville, FL, USA) [3]. Furthermore, alloplastic prostheses offer numerous advantages, including the immediate commencement of physical therapy, the elimination of the need for a secondary donor site, decreased operation time, and the ability to closely mimic normal anatomy. However, they also present certain disadvantages, including the cost of the device and the inability of alloplastic implants to accommodate the patient’s growth [4]. Moreover, some unwanted adverse effects may still occur with alloplastic TMJR, such as paralysis of the facial nerve branches, which accounts for 7.8% of all cases, followed by sensory alterations at 1.8%, heterotopic bone formation at 1.0%, and infection at a 0.7% incidence rate, respectively [5]. Alloplastic joints offer a more precise replication of the natural joint anatomy, allowing for the restoration of vertical dimension, the avoidance of donor site complications, reduced surgery time, and a decreased risk of recurrent ankylosis. These benefits enable immediate physiotherapy and rehabilitation, leading to enhanced outcomes for patients. However, the potential drawbacks of alloplastic reconstruction mainly revolve around material wear or failure, which can trigger adverse reactions like implant loosening and structural changes. Other concerns include long-term stability, costs, dystrophic bone development, and the unsuitability of these joints for children due to growth limitations. Comparative studies between autogenous and alloplastic TMJ reconstructions are limited, with few exceptions [2]. Indications of alloplastic prostheses include ankylosis or re-ankylosis with severe anatomic abnormalities, destruction of autogenous graft tissue by pathology, severe inflammatory joint disease, such as rheumatoid arthritis that results in anatomic mutilation of the joint component, and functional disability. The contraindications of these prostheses include the age of the patient, mental status of the patient, uncontrolled systemic disease, such as diabetes mellitus, active infection at the implantation site, and allergy to the materials that are used in the devices to be implanted [4]. Previous literature discussed alloplastic prostheses in the extended reconstruction of TMJ. However, there are no current comprehensive reviews broadly discussing classifications, indications, contraindications, virtual surgical planning, success criteria, and surgical approach to these alloplastic prostheses. Thus, this current literature review aims to discuss the utilization of alloplastic prostheses in the extended reconstruction of TMJ.

## 2. Materials and Methods

**Figure 1:**
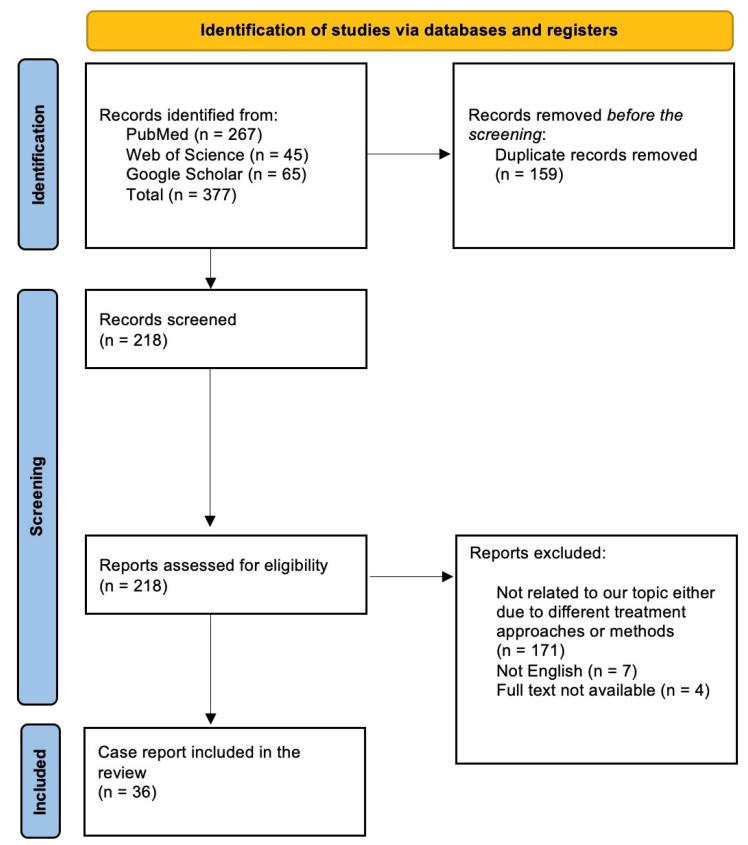
Study flowchart.

This review examines published studies on the use of extended alloplastic temporomandibular joints for jaw reconstruction. A comprehensive exploration of databases like PubMed, Web of Science, and Google Scholar using keywords, such as extended temporomandibular joint reconstruction, temporomandibular joint replacement, virtual surgical planning, computed tomography, and maximal interincisal opening, was performed to gather relevant literature. A comprehensive literature review was performed using three key databases: PubMed, Web of Science, and Google Scholar. Similar reviews in the field have also relied on these databases to ensure a robust selection of relevant studies. For instance, Zieliński et al. [6] conducted a systematic review that utilized these databases to explore the global prevalence of sleep bruxism and awake bruxism in pediatric and adult populations, while Mauro et al. [7] referenced the same databases to assess temporomandibular disorder management. By utilizing these databases, we aimed to ensure a diverse and comprehensive overview of the existing literature relevant to our research question. The selection of these databases was based on their unique strengths, which together facilitate a comprehensive approach to the literature search such as in the Mauro et al. study [7]. The inclusion criteria focused on reviews, clinical trials, and cohort studies with no time restrictions, while studies that were presented in a language other than English, had no full text available, or did not discuss alloplastic prosthesis in extended TMJ reconstructions were excluded. The acronym PICOS was used to answer this review question, “What are the uses of alloplastic prostheses in the extended reconstruction of TMJ?” PICOS stands for P = extended reconstruction of TMJ, I = Alloplastic prosthesis, C = Control group if applicable, and O = Outcomes. The search strategy was applied to PubMed, Google Scholar, and Web of Science without time restrictions. The search had no time-specific restrictions; the search period started in August 2024, and all papers were included until August 2024 with no time restrictions. Our review focused on all the relevant studies discussing extended alloplastic temporomandibular joint and their classifications, indications, contraindications, advantages, disadvantages, complications, virtual surgical planning (VSP), criteria for successful alloplastic devices, and the surgical approach. By using this process, all the papers discussing the utilization of alloplastic prostheses in the extended reconstruction of TMJ were obtained and included in our inclusion criteria. The studies that had poor methodological quality and insufficient data were excluded. Using the following search terms (extended alloplastic temporomandibular joint reconstruction, alloplastic temporomandibular joint replacement, total joint replacement, and total joint reconstruction), all papers discussing the utilization of alloplastic prostheses in the extended reconstruction of TMJ were gathered. In the initial stage, two reviewers (KM and BF) examined the titles and abstracts of the papers based on set criteria to choose articles for full reading. In the next stage, two reviewers (YA and KH) individually evaluated the selected articles based on the criteria for full reading. In case of any disagreement, discussion and consensus were reached to solve it, or a third reviewer (WM or SS) was consulted in cases of persistent conflict. The initial screening elicited 377 papers. After applying our inclusion criteria, the most relevant studies were selected and used in the current review. This review ultimately involved 36 papers discussing the use of extended alloplastic temporomandibular joints in the reconstruction of the temporomandibular joint. Figure 1 shows the study flowchart.

## 3. Literature Review

### 3.1. Classifications

**Figure 2:**
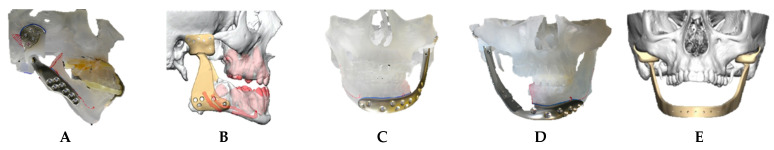
Illustrative image of mandible component in eTMJR classification (**A**) M0, (**B**) M1, (**C**) M2, (**D**) M3, and (**E**) M4 [1,3,10].

**Figure 3:**
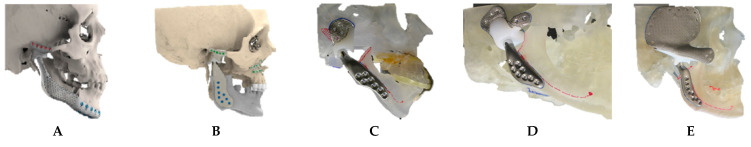
Illustrative image of fossa component in eTMJR classification (**A**) F0, (**B**) F1/FA, (**C**) F2/FA, (**D**) F3/FA, and (**E**) F4/FT [1,11].

For any classification system, certain rules must be abided by to ensure clarity and ease of usage, exhaustiveness, mutual exclusivity, clinical relevance, flexibility, and appropriateness across language barriers. The original classification of eTMJR was introduced in 2018 and later modified in 2021 [1,8]. In the original classification, it is divided into the fossa component (F0, F1, F2, F3, F4, and F5) and the mandible component (M0, M1, M2, M3, and M4). In the fossa component, F0 represents the standard fossa component, F1 represents anterior extension but does not surpass the articular eminence, F2 represents extension beyond the articular eminence anteriorly (zygomatic arch defect), F3 represents a temporal bone defect excluding the auditory apparatus with or without an arch defect, F4 represents a temporal bone defect involving the auditory apparatus with or without an arch defect, and F5 represents a temporal defect extending to the jugular foramen. Alternatively, in the mandible component, M0 represents the standard condyle-ramus component (proximal to the angle of the mandible), M1 represents extension proximal to the ipsilateral mental nerve foramen/region, M2 represents extension proximal to the contralateral mental nerve foramen/region, M3 represents extensive extension beyond the contralateral mental nerve foramen/region, and M4 represents a total alloplastic mandible (including both condyles) [1]. However, the modified classification embraces the same pattern as the original, wherein it is divided into the fossa component (F0, FA, and FT) and the mandible component (M0, M1, M2, M3, and M4). In the Fossa component, F0 is the same as the original, FA represents an extended fossa component reaching into the zygomatic arch, and FT represents an extended fossa component extending to cover a temporal bone defect. In the mandible component, M0, M1, M2, M3, and M4 are the same as the original classification [8,9]. Table 1 shows a comparison between the original and modified classification of eTMJR [8]. Even though the eTMJR classification is based on the length of the prosthesis in the mandible and the fossae component, the width of the prosthesis was not considered in both classification systems, which raises some limitations in the original and modified versions [3]. Khattak et al. examined the use of eTMJR prostheses in treating large craniomaxillofacial defects. The analysis included patient baseline characteristics, treatment outcomes related to functional variables, and complications post-eTMJR prosthesis implantation. The study found that eTMJR prosthesis implantation was infrequent, lacked specification on the appropriate class of eTMJR prosthesis, and noted significant variations in the width of the mandibular component of eTMJR prostheses [3]. Figure 2 shows an image representing the mandible component in eTMJR classification [1] (Figure 2A,C,D), [3] (Figure 2B), and [10] (Figure 2E). Furthermore, Figure 3 displays an image representing the fossa component in eTMJR’s original and modified classifications [11] (Figure 3A,B), and [1] (Figure 3C–E).

Management of eTMJR can be classified based on the timing of treatment into immediate primary TMJR, delayed primary TMJR, secondary TMJR, or delayed secondary TMJR. Immediate primary TMJR is a one-stage procedure that requires a mandibular resection with disarticulation of the condyle to address a pathological process. Cases that would benefit from this type of treatment include benign pathologies such as ameloblastoma or malignant pathologies that do not require post-operative radiation, such as osteosarcoma, and extremely severe bone resorption, as can be seen in the scleroderma. Delayed primary TMJR is indicated for patients who previously underwent treatment for a primary pathology and immediate reconstruction was contraindicated, such as cases involving osteomyelitis or avulsive trauma. Secondary TMJR is used for patients whose defects were immediately reconstructed by placing a condyle-supported metallic reconstruction plate directly against the mandibular fossa. It serves as an interim solution to avoid complications such as mandibular dysfunction, hardware failure, or displacement into the medial cranial fossa or the external auditory canal until the patient-specific prosthesis design is completed, approved by the surgeon, and fabricated. Delayed secondary TMJR is performed in patients who have undergone multiple prior autogenous or alloplastic reconstructions that have failed due to unsatisfactory outcomes caused by hardware failure [12].

### 3.2. Indications and Contraindications

Indications for eTMJR include posttraumatic damage or loss of the mandible or condyle, mandibular or TMJ tumors, autoimmune or connective tissue diseases, mandibular or TMJ osteomyelitis, and past unsuccessful alloplastic reconstructions [12]. Furthermore, based on United Kingdom guidelines for TMJ replacement, at least two of the following criteria should be met to assess the outcomes: a dietary score of <5/10 (liquid score 0 and full diet scores 10); restricted mouth opening (less than 35 mm); occlusal collapse (retrusion or anterior open bite); loss of height of vertical ramus and severe condylar resorption; pain score >5 out of 10 on the visual analog scale; and further quality of life issues [13].

The contraindications of eTMJR include uncontrolled systemic disease, mental instability, active infection, allergies to prosthetic components, young growing patients, and uncontrolled parafunctional habits [4,12].

### 3.3. Virtual Surgical Planning

Virtual surgical planning (VSP) has revolutionized the field of oral and maxillofacial surgery by streamlining and enhancing the precision of case preparation. It enables surgeons to implement precise surgical strategies for offering patients optimal long-term functional and esthetic results for complicated reconstructive interventions like eTMJR [14]. Recent advancements in VSP have allowed surgeons to integrate Computed Tomography (CT) scan digital images into a unified 3D planning software, segmenting the CT scan into mandible and skull components. Specific bony areas or regions for planned removal are isolated, and additionally, intraoral scans are meticulously aligned and combined using occlusion data from the CT scan. By establishing a cutting plane, simple resections can be identified, while complex curved osteotomies are delineated through freehand marking. Each patient receives a personalized simulation approach tailored to their specific condition. Once the VSP and the customized prosthesis design are approved by the surgeon, the production of the surgical template guide and 3D metal prosthesis, encompassing fossa and mandible elements is initiated [11,15].

### 3.4. Criteria for Successful Alloplastic Device

The alloplastic device must be biocompatible, with viable selections encompassing titanium, cobalt–chromium, cobalt–chromium–molybdenum, and ultra-high molecular weight polyethylene, all of which boast a longstanding history of application in orthopedic joint replacements. Furthermore, the device must exhibit resilience against the forces exerted across the complete spectrum of joint motion. Additionally, it is imperative for the device to maintain stability in situ during the implantation process. Lastly, the implantation procedure for the alloplastic device must be executed within an aseptic setting [4].

### 3.5. Surgical Approach

**Figure 4:**
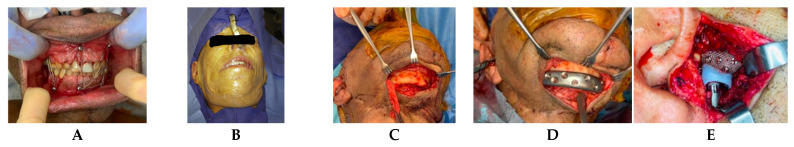
Surgical approach for eTMJR. (**A**) Occlusion is stabilized using IMF screws and wires. (**B**) Ioban isolation after draping. (**C**) Dissection from the submental region. (**D**) Fixation of the mandible component with alloplastic device. (**E**) Fixation of the fossa component with alloplastic device [16].

Once the patient is brought to the operating room awake and in the supine position, monitors will be connected, general anesthesia induced, intubation performed, and the patient will be prepared and draped in a sterile manner. Moreover, the face, preauricular area, and neck will be exposed bilaterally. First, occlusion will be stabilized in the centric relation using Intermaxillary Fixation (IMF) screws and wires, as shown in Figure 4A [16]. Furthermore, Ioban (3M) will be used to isolate the mouth, nose, hairline, and endotracheal tube for the sterile field, as seen in Figure 4B [17]. Consequently, surgical markings will be made in the periauricular and submandibular or submental region depending on the extent of the defect in the mandible component.

In the periauricular region, an endural incision will be performed through the skin. Depending on the fossa component, a temporal extension might be made in cases of F3/FA, F4/FT, or F5/FT. Dissection progresses from subcutaneous tissue toward the temporoparietal facia and is directed toward the temporalis muscle facia through the avascular plane, moving down to the joint capsule. Subsequently, the zygomatic arch will be identified, and subperiosteal dissection will be made to protect the temporal branch of the facial nerve.

In the submental region, an incision following the curvature of the anterior mandible exposes symphyseal and parasymphyseal areas. Following the incision, dissection from subcutaneous tissue progresses toward the platysma and reaches the pterygomasseteric sling to access the ramus as seen in Figure 4C [16]. Afterward, a tunnel is created to have communication between the two spaces for the alloplastic implantation device.

Depending on the mandible component, the extent of the defect or disease will determine how much resection is required that has been pre-planned in VSP; this means a condylectomy will be performed in cases of M0, a condylectomy with coronoidectomy for M0, a horizontal ramus osteotomy for M0/M1, a hemi-mandibulectomy for M2/M3, or a total-mandibulectomy for M4. Fossa will be debrided from all soft tissue. A VSP-designed surgical cutting guide will be inserted, and osteotomy will be performed for all segments, starting from the most distal zones. Irrigation with an antibiotic solution is done after the osteotomy is completed, and defective bone is removed prior to alloplastic device implantation.

Finally, after the alloplastic device designed by VSP is implanted, and once it is stable and in situ, as reflected in Figure 4D,E [16], closure of the surgical site will be done using sutures in a layered fashion [14,16,17].

## 4. Discussion

The future of the extended alloplastic temporomandibular joint reconstruction appears to be promising in providing improved outcomes of jaw reconstruction, offering enhanced precision and customization through Virtual Surgical Planning (VSP), and potentially increasing patient comfort and functionalities. The advantages of the Extended Alloplastic Temporomandibular Joint include superior jaw reconstruction results, improved precision and customization through Virtual Surgical Planning, and potentially improved functional outcomes in patients. According to our current review, the Extended Alloplastic Temporomandibular Joint (eTMJR) is an advanced method for treating complex TMJ issues and related mandibular or skull base abnormalities. This technique offers significant improvements in function and appearance by using VSP advancements and various biocompatible artificial materials, representing a notable advancement in TMJR treatments. Compared to natural grafts, alloplastic implants yield better results in jaw movement and pain control, highlighting their potential as a preferred treatment option. Historically, concerns have been raised regarding the long-term stability of alloplastic prostheses, especially the possibility of material wear and failure over time [6]. Contrary to this claim, a retrospective study by Wolford et al., which included 56 patients treated with alloplastic TMJR for end-stage TMJ disease over a 20-year follow-up, compared pre-operative and post-operative Maximal Incisal Opening (MIO) in millimeters, pain levels (rated on a numerical scale from 0 being no pain to 10 being the worst pain imaginable), jaw function (rated on a numerical scale from 0 being normal to 10 being no jaw movement), diet (rated on a numerical scale from 0 being no restriction to 10 being liquids only), and post-surgical quality of life (categorized as improved, same, or worse). Overall, the study demonstrated that alloplastic devices continue to function well and are unlikely to fail due to material wear [18]. Despite its advantages, the Extended Alloplastic Temporomandibular Joint may be associated with limitations such as high cost, potential risks of infection or implant failure, and complications like malpositioning, discomfort, or the need for additional surgeries to address subsequent issues. Regarding alloplastic eTMJR, a comprehensive analysis by Khattak et al. (2023) reviewed 49 cases managed with eTMJR prostheses, including 27 different studies drawn from the literature with follow-up durations ranging from 4 to 84 months. The studies compared pre-operative and post-operative MIO in millimeters, occlusion (categorized as malocclusion, stable, good, or excellent), symmetry (categorized as stable, adequate, good, or excellent facial asymmetry), pain (rated on a numerical scale from 0 to 10), and diet (categorized as liquid, soft, restricted, or normal). In terms of post-operative MIO, 17 out of the 49 cases did not provide assessable numerical data. Of the 32 cases that did, 19 reported MIO greater than 35 mm, while the remaining 13 reported an average MIO of 27.5 ± 6.5 mm. For post-operative occlusion, 19 cases did not provide assessable categorical data. Among the 30 assessable cases, nine reported excellent occlusion, four reported good occlusion, and 17 reported stable occlusion. Regarding postoperative symmetry, 23 cases did not provide assessable categorical data. Of the 26 assessable cases, two reported excellent symmetry, 14 reported good symmetry, five reported adequate symmetry, and five reported stable symmetry. Concerning postoperative pain, 30 cases did not report pain scale scores. Of the 19 cases that did, five reported an average pain scale score of 1.7 ± 0.4, 13 reported a pain scale score of 0/10, and 1 reported a score of 1/10. For post-operative diet, 33 cases did not provide assessable categorical data, while all 16 of the remaining cases reported a normal diet [3]. Similarly, a retrospective study was conducted on 99 patients, comparing 50 alloplastic and 49 autogenous costochondral graft cases in TMJR with a follow-up duration of at least 24 months. The study compared pre-operative and post-operative MIO, pain, and diet. For MIO, the alloplastic group showed an increase from a pre-operative mean of 17.1 mm to a post-operative mean of 25.2 mm, while the autogenous group showed a modest change from 23.2 mm pre-operatively to 24.6 mm post-operatively. Regarding pain, the alloplastic group reported a decrease in pain from 6.7 pre-operatively to 2.4 post-operatively, whereas the autogenous group showed a reduction from 7.6 pre-operatively to 3.8 post-operatively. In terms of diet, represented numerically (1 = liquid diet, 2 = soft, 3 = restricted, and 4 = normal), the alloplastic group improved from a mean of 2.0 pre-operatively to 3.6 post-operatively, while the autogenous group exhibited improvements from 2.3 pre-operatively to 3.0 post-operatively [2]. Elledge et al. [19]. conducted a thorough review of data from the British Association of TMJ Surgeons National Case Registration of TMJ Replacement. They analyzed 252 one-year outcome records, showcasing significant improvements in interincisal distance and pain scores. The majority of patients observed positive changes, with notable enhancements in quality of life. This national database in the UK marks a significant step in tracking the long-term outcomes of TMJ replacements, offering promising results and plans for future data analysis [19]. Idle et al. aimed to collect long-term data on TMJ replacement across UK centers. They focused on 402 patients who underwent TMJ replacement between 1994 and 2012, with the majority being female. The study included various diagnoses leading to joint replacement. The study further discussed preoperative conditions, surgical techniques, and intends to track patient outcomes over time [20]. Westermark et al. detailed four cases where TMJ reconstruction was carried out using custom TMJ concepts joint prostheses. These prosthetic components were tailored to address significant defects in the zygomatic arch, mandibular ramus, and body of the mandible, with one instance of the mandibular component being used to restore total mandibular continuity. These prosthetic components facilitated excellent anatomical reconstruction and served as a feasible treatment option when autogenous grafts were not suitable due to underlying pathological conditions. The prostheses have been functioning effectively for up to six years. However, in one case, revision surgery was needed due to the absence of a pterygomasseteric sling that led to the condyle slipping out of the fossa. Based on the authors’ clinical observations, utilizing a customized prosthesis in conjunction with TMJ reconstruction appears to be a dependable treatment alternative for addressing intricate, significant maxillo-mandibular defects [21]. Ruiz et al. described two cases of sizeable solid/multicystic ameloblastomas treated with hemimandibulectomy and reconstructed using a unique method involving a custom TMJ Concepts prosthesis. The report explores the impact and results of custom temporomandibular joint prostheses in such cases [22]. Moreover, Morrison et al. detailed a case involving a recurrent ameloblastoma managed through left hemimandibulectomy and prompt reconstruction using a personalized alloplastic system along with an anterior iliac crest bone graft. The outcome showcased significant mandibular function and facial esthetics, minimal issues at the donor site, and a swift return to normal function [23].

Alloplastic reconstruction techniques have become increasingly popular, showing success in treating various conditions like ankylosis, condylar fracture, extensive resection neoplasia, severe inflammatory TMJ disease, and congenital TMJ abnormalities. Horen et al. discussed a case where a patient underwent successful ameloblastoma resection and TMJ reconstruction using a custom TMJ Concepts alloplastic implant. Horen et al. provided a literature review on alloplastic TMJ reconstruction post-ameloblastoma resection [24]. In cases of severe temporomandibular joint issues, surgical reconstruction is often necessary to restore function and anatomy. While some patients can be treated with their own tissue, complex reconstructions may require the use of alloplastic materials. Ramil et al. reported details of four clinical cases where either autologous grafts or Christensen joint prostheses were used for temporomandibular joint reconstruction [25]. Jones et al. reported on seven patients who underwent a total of 12 joint replacements using the TMJ Concepts system or the Biomet Lorenz joint system. Two patients (three joints) received the TMJ Concepts system and five patients (nine joints) received the Biomet Lorenz system. Despite being early, the outcomes were generally positive, with the longest replacement in place for three years and the most recent for six months. On average, postoperative mouth opening was 29.7 mm (range: 25–35 mm) with an average pain score of 1.7 (range: 0–3). Complications were minimal, including sensory disturbance to the lip in one patient and joint dislocation in two patients [26]. Mercuri et al. suggested that using a patient-fitted prosthesis for total alloplastic replacement can effectively manage reankylosis of the TMJ. Additionally, autogenous fat transplantation may help reduce the recurrence of joint calcification, leading to improved and consistent mandibular motion [27].

The primary aim of TMJ total joint replacement is to restore mandibular function and form. Data show that pain relief, while beneficial, is not the main focus. Despite some lingering chronic pain, improvements in mandibular function and form have enhanced the quality of life for 85% of long-term custom TMJ total joint replacement patients. Mercuri et al. advocated for the use of custom TMJ total joint replacement devices as a fitting solution for end-stage TMJ disorders, aligning with orthopedic criteria for successful joint replacement [28]. Landes et al. presented two cases involving different reconstruction needs. The first patient underwent mandibular body and ascending ramus reconstruction following sarcoma resection, initially using a condyle-bearing reconstruction plate that led to significant dysfunction. Subsequent treatment involved a one-stage vascularized iliac crest free flap and an alloplastic TMJ prosthesis for mandibular reconstruction, along with metal removal, soft tissue augmentation through lipotransfer, and dental implant placement. After 63 months, the patient showed improvements in pain, mouth opening, protrusion, and lateral excursion. In the second case, mandibular body, ascending ramus, and joint reconstruction were carried out using a transoral vascularized fibula free flap with temporal vessel anastomosis. This corrected a traumatic deep bite and facial height issues, avoiding additional submandibular scars through transoral placement of the fibula transplant. A miniaturized TMJ prosthesis was also used alongside the vascularized free flap, with a 28-month follow-up [29]. Worrall et al. noted that some patients with birth-related anatomical deficiencies, like cleft lip and branchial arch syndrome, do not always receive the necessary surgical corrections. Despite attempts at reconstructing missing mandibular and TMJ structures using autogenous methods, some patients experience relapses into more severe and long-lasting conditions. Utilizing a Christensen Fossa-Eminence Prosthesis (FEP) and Condylar Prosthesis (CP) within a total joint replacement solution can lead to improved TMJ mobility, pain reduction, and the mechanical replacement of missing anatomical structures with predictable and enduring results. Additionally, this approach can enhance the patient’s esthetics [30]. Hodzic et al. [31] conducted a retrospective study at Helsinki University Hospital over the last decade, focusing on TMJ reconstruction using alloplastic prostheses for patients with congenital syndromes. The study included seven patients with a total of ten treated joints affected by syndromes like Goldenhar syndrome, hemifacial macrosomia, Nager syndrome, and Treacher-Collins syndrome. Most patients had previous facial skeletal operations in childhood, which may have impacted the outcomes of the TMJ surgery. Results showed successful postoperative mouth opening with an average of 34 mm (range 24–42 mm). Alloplastic prosthesis for TMJ reconstruction provides new tools to address facial deformities in syndromes with craniofacial abnormalities, improving jaw function, symmetry, and mouth opening [29]. Sinn et al. found that using alloplastic material does not hinder mandible growth or impede improved maximum interincisal opening (MIO) based on our long- and short-term findings. These results show significant enhancements in MIO for patients with complex craniofacial deformities and traumatic injuries. The authors suggest that alloplastic joints offer a reliable way to restore MIO and potentially avoid the need for multiple, more challenging, and less successful surgeries [32]. Goker et al. studied a new custom-made prosthetic system in a 12-year-old patient with TMJ ankylosis and hypoplasia. The patient had previously undergone two autogenous graft surgeries. The right side of the face showed swelling and tumefaction. The patient had a 1.5 cm mouth opening with limited jaw movements. Muscle hypertonicity and palpation pain were noted. No signs of dislocation, fracture, or traumatic damage were observed. TC scans revealed unilateral TMJ ankylosis. Revision surgery was planned using a customized plastic temporomandibular joint prosthesis. The patient received a TMJ reconstruction with a patient-specific CADCAM custom-made prosthesis, leading to successful healing without complications during the 46-month follow-up. Goker and colleagues studied a new custom-made prosthetic system in a 12-year-old patient with TMJ ankylosis and hypoplasia. The patient had previously undergone two autogenous graft surgeries. The right side of the face showed swelling and tumefaction. The patient had 1.5 cm mouth opening with limited jaw movements. Muscle hypertonicity and palpation pain were noted. No signs of dislocation, fracture, or traumatic damage were observed. TC scans revealed unilateral TMJ ankylosis. Revision surgery was planned using a customized plastic temporomandibular joint prosthesis. The patient received TMJ reconstruction with a patient-specific CADCAM custom-made prosthesis, leading to successful healing without complications during the 46-month follow-up [33]. Adhikari et al. detailed the case of a 47-year-old woman with a 30-year history of complete jaw immobility, difficulty chewing and speaking, and reliance on a liquid diet. Bilateral TMJ ankylosis and a nine mm deviation of the chin to the right were identified. Surgery involved bilateral osteoarthectomy, reconstruction of the TMJ with a custom alloplastic prosthesis, utilizing the abdominal fat pad to prevent recurrence. Genioplasty adjusted the chin by nine mm to the left through a vestibular approach. After surgery, the patient achieved a 30 mm mouth opening, and facial asymmetry from the chin deviation was corrected [34]. Yadav et al. outlined the role of alloplastic total joint prosthesis in replacing the TMJ. They emphasized that total joint replacement is a common procedure for advanced TMJ disease, underscoring the importance of all maxillofacial surgeons being familiar with TMJ replacement [35]. Roychoudhury et al. aimed to discuss the role of alloplastic total joint replacement (TJR) in treating TMJ ankylosis (TMJA). The current evidence strongly supports the effectiveness of alloplastic TJR in managing TMJA, leading to notable improvements in mouth opening, facial symmetry correction, reduced recurrence, and enhanced quality of life. TMJ TJR is increasingly becoming the preferred treatment for TMJA, although cost considerations may limit access to this treatment option at times [36]. The study by Bhargava et al. aimed to assess the potential of a personalized alloplastic Temporomandibular Joint device in patients undergoing total temporomandibular joint reconstruction. The findings indicate that this customized alloplastic joint replacement is a safe, effective, and dependable option for treating patients with severe TMJ dysfunction necessitating total joint reconstruction [37]. The study by Bhargava and colleagues aimed to explore the clinical effectiveness of a hybrid alloplastic temporomandibular joint prosthesis in patients undergoing total alloplastic joint replacement for the TMJ. The prosthesis used in the study was a hybrid version combining a stock prototype design with partially customized components. The study findings revealed improvements in functional and observational outcomes during post-operative follow-up compared to pre-operative assessments. Patients experienced enhanced overall quality of life and nutritional status after the operation. The follow-up period exhibited subjective and objective enhancements in the evaluated parameters among the study participants [38]. According to Jones et al. [26] when evaluating complications related to alloplastic prostheses, they explained that three patients experienced minor complications. One of them, a 75-year-old female with severe osteoarthritis, developed bilateral paresthesia of the inferior dental nerves post-surgery due to improper placement of the ramus component of the prosthesis, leading to a Class II occlusion after the operation. The paresthesia is gradually improving. The other two patients suffered mandibular condyle dislocation from the glenoid fossa shortly after surgery, necessitating relocation under general anesthesia and using intermaxillary elastics for stabilization [26]. The seamless integration of custom TMJ TJR components with the host bone and stable screw fixation allows for immediate mandibular function post-implantation. This early restoration is crucial in severe joint diseases where muscle function is already compromised, making delayed physical rehabilitation challenging. Salter’s research emphasizes the significance of prompt active physical therapy for optimal long-term outcomes post-joint surgery [28]. Potential adverse outcomes of alloplastic temporomandibular joint replacement include continued or increased pain levels or worsening of TMJ symptoms, infection, and heterotopic bone formation [28]. Future advancements in technology and materials may lead to further enhancements in Extended Alloplastic Temporomandibular Joint procedures, aiming to address current limitations, improve outcomes, and potentially expand its applicability in jaw reconstruction surgeries.

This paper’s limitations include the decision to employ a traditional literature review format, which restricts the depth and rigor typically afforded by systematic reviews. To strengthen future research in this area, we recommend that subsequent reviews adopt systematic analyses and consider the possibility of conducting a meta-analysis. Such approaches would enhance the robustness of findings and support more conclusive insights into the topic.

## 5. Conclusions

In conclusion, eTMJR emerges as a cutting-edge approach for addressing complicated TMJ issues and associated mandibular and/or skull base anomalies. By leveraging the advancements in VSP and the wide variety of biocompatible alloplastic materials, this approach promises enhanced functional and esthetic results and signifies a significant leap forward in the realm of TMJR interventions. To further solidify the evidence supporting the efficacy of eTMJR in treating extensive and intricate craniomaxillofacial defects, additional retrospective studies focusing on the longitudinal survival of these prostheses are warranted to comprehensively evaluate its long-term effectiveness and patient outcomes. These retrospective investigations can elucidate different methods for the evaluation of the outcome and effectiveness of the prosthesis using a thorough and continuous evaluation by the clinician.

## Figures and Tables

**Table 1:** Comparison between original and modified classifications of eTMJR [11].

Component	Original Classification	Modified Classification
**Fossa**	F0	F0
	F1	FA
	F2	
	F3	
	F4	FT
	F5	
**Mandible**	M0	M0
	M1	M1
	M2	M2
	M3	M3
	M4	M4

## Data Availability

All articles cited in this review are available on PubMed and Google Scholar.
